# Exploration of Gender-Sensitive Care in Vocational Rehabilitation Providers Working With Youth With Disabilities: Codevelopment of an Educational Simulation

**DOI:** 10.2196/23568

**Published:** 2021-03-15

**Authors:** Sally Lindsay, Kendall Kolne, Donna J Barker, Angela Colantonio, Jennifer Stinson, Sandra Moll, Nicole Thomson

**Affiliations:** 1 Bloorview Research Institute Holland Bloorview Kids Rehabilitation Hospital Toronto, ON Canada; 2 Department of Occupational Science & Occupational Therapy University of Toronto Toronto, ON Canada; 3 Lawrence S. Bloomberg Faculty of Nursing University of Toronto Toronto, ON Canada; 4 Hospital for Sick Children Toronto, ON Canada; 5 School of Rehabilitation Sciences McMaster University Hamilton, ON Canada; 6 Centre for Addiction and Mental Health Toronto, ON Canada

**Keywords:** continuing education, gender-identity, gender-sensitive care, rehabilitation

## Abstract

**Background:**

Although research shows that there is a need for gender-specific vocational support to help youth with disabilities find employment, health care providers often report needing more training in this area. Currently, there are no existing educational simulations of gender-sensitive care within vocational rehabilitation for clinicians who provide care to youth with disabilities. Therefore, developing further educational tools that address gender-sensitive care could help them enhance the care they provide while optimizing patient outcomes.

**Objective:**

This study aims to codevelop an educational simulation and identify issues relevant to providing gender-sensitive care within the context of vocational rehabilitation for youth with disabilities.

**Methods:**

We used a qualitative co-design approach with a purposive sampling strategy that involved focus group discussions and journal reflections to understand and address issues relevant to gender-sensitive care within vocational rehabilitation for those working with youth with disabilities. A total of 10 rehabilitation providers participated in two sessions (5 participants per session) to design the web-based simulation tool. The sessions (2.5 hours each) were audio recorded, transcribed, and analyzed thematically.

**Results:**

Two main themes arose from our analysis of codeveloping a simulation focusing on gender-sensitive care. The first theme involved the relevance of gender within clinical practice; responses varied from hesitance to acknowledging but not talking about it to those who incorporated gender into their practice. The second theme focused on creating a comfortable and safe space to enable gender-sensitive care (ie, included patient-centered care, effective communication and rapport building, appropriate language and pronoun use, respecting gender identity, awareness of stereotypes, and responding to therapeutic ruptures).

**Conclusions:**

Our web-based gender-sensitive care simulation that addressed vocational rehabilitation among youth with disabilities was cocreated with clinicians. The simulation highlights many issues relevant to clinical practice and has potential as an educational tool for those working with young people with disabilities.

## Introduction

### Background

Within Canada, there are more than 200,000 youth aged between 15 and 25 years who have a disability [1]. Such youth often encounter significant challenges in finding employment, including discriminatory attitudes, inadequate transportation, and inaccessible workspaces [2,3]. Consequently, youth with disabilities often have higher unemployment rates than those without disabilities [4]. Focusing on the youth is salient because many disadvantages are often compounded for those who start life with a disability or acquire it at an early age [5,6]. For example, youth with disabilities often encounter challenges with developmental tasks, social development, and role functioning [5,7]. In addition, this developmental period is characterized by identity exploration, instability, and continued development of executive functioning, which is important for enhancing job skills and independence [5]. Furthermore, young adulthood is a critical time for optimizing work-based identities and positive behaviors [2,7]. Unfortunately, youth with disabilities often do not receive the appropriate support they need to find meaningful employment. For example, even though clinicians who work with such youth acknowledge gender differences within their practice, many admit to not providing appropriate or gender-specific support to help them with their vocational goals [8]. Furthermore, recent research shows that sex-specific vocational support is needed to help youth as they transition into employment roles [8,9]. Therefore, further training tools are needed to help clinicians provide gender-sensitive care in vocational rehabilitation for youth with disabilities.

### Gender and Vocational Rehabilitation for Youth With Disabilities

Gender plays an important role within vocational rehabilitation for youth with disabilities. Research shows that gender often shapes how people with disabilities cope with engaging in vocational training and employment [8-12]. For example, a recent systematic review on the role of gender in employment among youth with disabilities found that young women with disabilities continue to lag behind their male peers on several health and social outcomes, including lower employment rates and multiple forms of discrimination [8,9,13,14]. Young women with disabilities often encounter particular barriers in career development, including limited vocational training, lower family expectations, disability stereotypes, and decreased self-confidence [15,16]. Although gender is becoming increasingly important, the intersection of disability and youth employment has received little attention [17]. Understanding this intersection is critical because inequalities in employment are significant for individuals with disabilities who identify as men, women, gender fluid, or nonbinary [18].

Of the limited research exploring gender and employment among youth with disabilities, the focus is often on employment outcomes (eg, working or unemployed) or pay differences [8,9]. Such studies also tend to only look at gender from a binary (ie, man or woman) perspective, which is problematic because it does not capture the diversity of gender identity experiences. Thus, there is a need for further research to unpack the complex relationship between gender, vocational rehabilitation, and employment.

Although there are several systematic reviews focusing on youth with disabilities and employment, there is very little, if any, mention of gender [13,17,19-21]. Meanwhile, several studies highlight the important need for gender-specific vocational support for youth with disabilities [13,22-24]. Exploring gender within the context of vocational rehabilitation for youth with disabilities is important for decision making, enhanced communication, and engagement in programs and interventions [25].

### Gender-Sensitive Care

Despite the importance of gender (ie, socially constructed roles, behaviors, and identities) within health care, many health care providers report that they lack knowledge on how to provide gender-sensitive care (ie, knowledge and competence about the role of gender within clinical practice) [26], including within rehabilitation settings [27]. Such a shortage of knowledge in this area is concerning because it can influence health outcomes, health inequalities, incorrect or delayed diagnoses, and suboptimal therapies [28-30].

Gender-sensitive approaches aim to incorporate specific health care needs of men and women while aiming to address gender-based health inequities and methods of transforming harmful gender norms, roles, and relations while also centering on promoting gender equality [31]. Gender sensitivity involves understanding gendered patterns of individual experiences of boys and men as well as girls and women and the unique experiences of those who have identities that are nonbinary or gender fluid; it also considers their morbidity and mortality while reflecting on the broader sociopolitical and cultural context of where health care takes place [26,32]. Although research on gender-sensitive care is increasing, it mainly focuses on adult and acute care populations, with little mention of pediatrics or rehabilitation, especially within the context of vocations [33].

Research consistently highlights that gender plays a critical role in the incidence, clinical presentation, manifestation, and health outcomes; yet, youth with disabilities are often viewed and treated as without gender and asexual [34-38]. For example, youth who identify as lesbian, gay, bisexual, transgender, or queer (LGBTQ+) have been largely ignored in vocational rehabilitation research [8,26,28]. This trend is concerning because sexual and gender minority groups often encounter substantial barriers within the health care system including discrimination, which could affect their health and vocational outcomes [39-44].

A recent review on gender-sensitive educational interventions for health care providers found that few studies focused on rehabilitation care providers and none focused on vocational rehabilitation for youth with disabilities. Currently, there are no existing gender-sensitive care educational simulations within vocational rehabilitation for clinicians who provide care to youth with disabilities [28]. Therefore, developing further educational tools in gender-sensitive care could help them enhance the care they provide while optimizing patient outcomes.

### Simulations as a Professional Development Tool

There is currently a lack of consensus regarding the best educational approaches to teach gender-sensitive care [45,46]. One potential way to address the need for further education to health care providers is through simulations, which refer to life-like environments and contrived social situations mimicking problems or conditions arising in professional encounters [47]. Simulations are a critical tool for interprofessional training that can help health care providers enhance their clinical skills, teamwork, and communication [48,49]. Research highlights that simulation-based learning can be a positive educational experience that enhances patient outcomes [50-52]. Although health care providers frequently participate in simulations, they are rarely engaged in the design and development process [47,53,54]. Including health care providers in building a simulation, as opposed to just watching or participating in one, could also help to create relevant scenarios [53,55] while reinforcing clinical skills [47]. This study aims to involve health care providers in cocreating simulations.

Our simulation is novel because it was informed by several needs assessments from both health care providers and youth perspectives and is one of the first to explore gender-sensitive care among vocational rehabilitation care providers who work with youth with disabilities [56]. Developing further training for health care providers could help address gender-based health inequalities [46].

### Objective

The objective of our study was to codevelop an educational simulation and identify issues relevant to providing gender-sensitive care within the context of vocational rehabilitation for youth with disabilities.

## Methods

### Design

We used an interpretive descriptive qualitative design, which is particularly valuable in applied clinical health studies [57]. We conducted focus group discussions and participant journal reflections to understand participants’ experiences and perspectives of building the simulation. This design was appropriate because it is consistent with the development of professional simulation tools [49,53].

### Sample and Recruitment

This study was conducted at an academic pediatric rehabilitation hospital located in a large urban center where we received institutional ethics approval. All participants provided written consent before taking part following approval by the local research ethics board. Using a purposive sampling strategy, participants were recruited through invitation letters, referrals, or advertisements at a pediatric rehabilitation hospital. Participants who were interested were screened to meet the following eligibility criteria: a health care provider, practitioner, or trainee who had relevant experience in helping young people with disabilities to find employment. A total of 10 participants (9 women and 1 man) participated in the simulation build exercises (5 in each build session; Table 1). Note that not all participants took part in both sessions. We recognize that the gender composition of most of the participants in this sample is female; however, this is consistent with the gender composition of pediatric rehabilitation care providers [58]. Our sample size aligns with recommendations for the optimal size for conducting simulations and focus group methodology [59,60].

**Table 1:** Overview of participants.

Participant	Profession
1	Occupational therapist
2	Occupational therapist
3	Social worker
4	Social worker
5	Social worker
6	Vocational rehabilitation counselor
7	Vocational rehabilitation counselor
8	Occupational therapist
9	Vocational rehabilitation counselor
10	Occupational therapist

### Procedure for Developing the Web-Based Simulation

The simulation codevelopment sessions occurred over two 2.5-hour sessions, held in March and November 2019, and were facilitated by researchers (ie, the first two authors, SL and KK) who were certified in *SIM-one* simulations (ie, briefing, debriefing, and facilitating). The first session (n=5) focused on building the simulation scenario content, whereas the second session (n=5) piloted the scenario with live actors (ie, simulated participants) who were trained for their character roles before participating in the session. The authors had no previous relationship with the study participants.

#### First Build Session

The initial discussion with participants was informed by needs assessments and systematic reviews conducted by our team, focusing on the role of gender among youth with disabilities [8,9,13,17,61-64] and health care providers [13,64-66]. The rationale for this was to share evidence-informed, first-hand experiences. In particular, our discussion addressed research evidence about gender-sensitive approaches to engaging youth in rehabilitation (eg, rapport development, gender role expectations, self-advocacy, disclosure decisions) and self-reflection on gender in clinical practice, in addition to the lived experience perspectives of young people with disabilities. After giving participants an overview of the topic, we led a focus group discussion that allowed participants to reflect on their own clinical experiences regarding gender and share relevant issues.

Next, we gave participants a brief orientation on the process of building a simulation, which was led by a professionally trained simulation educator. We then followed a simulation scenario template (Multimedia Appendix 1), which guided participants through the learning objectives for building a simulation. Then participants were asked to help develop the content for the simulation by using a template, while a simulation educator and researchers guided a discussion about what the content would entail. Participants shared their experiences of how they addressed their gender in their practice. After the completion of each simulation, we had a focus group discussion (ie, simulation debrief) [59] (Multimedia Appendix 2 [53]). Participants then shared their reactions to the simulation content, its relevance to health care providers, and any recommendations they had for further development. After the first simulation session, the researchers then worked with a simulation educator and standardized participants to finalize a scenario script. We also met with a youth who has a disability and who also identified as transgender, and they provided feedback on our scenario, which we incorporated into the second build session.

#### Second Build Session

In the second simulation build session, we described the scenario template that the participants built in the first session. This session involved simulated participants who were trained for their character roles and then piloted the simulation with feedback from the health care provider participants. We then piloted the disability disclosure scenario, with feedback from participants through a guided focus group discussion (Multimedia Appendix 2). Next, we incorporated all participant feedback into the final version of the simulation, which will be filmed for further professional development purposes.

### Data Analysis

Both simulation codevelopment sessions were audio recorded, transcribed verbatim, and verified by the researchers who attended the simulation sessions. Two researchers independently reviewed the transcripts and developed the codes using an open-coding thematic approach [67]. The research question guided the analysis, where we looked for codes regarding gender-sensitive care. This approach involved the first two authors (with backgrounds in pediatric rehabilitation and gender-sensitive care) independently reading the transcripts and familiarizing themselves with the data, generating initial codes, and revising and defining the themes. We developed a list of preliminary codes while noting the patterns between them and then met to compare the codes. During this discussion, we split, merged, and relabeled codes until we reached an agreement in the coding tree. Team discussions also helped resolve any discrepancies in the organization of the themes. We checked for thematic saturation and felt that this was achieved in our codes and themes [67,68]. The first author, with experience in qualitative research, applied the final coding scheme to the transcripts. Relevant quotes that reflected each theme and subtheme were extracted [67]. Strategies to enhance rigor and trustworthiness of the findings included prolonged engagement, descriptive participant accounts, and peer debriefing discussions among the research team who have expertise in rehabilitation, pediatrics, and gender [68]. We also kept notes on key decisions made throughout the data analysis. The research team also reflected on their own biases and assumptions and interests in this topic and how this may have influenced their selection of the themes, which we noted in our audit trail [67,68].

## Results

### Overview

Two main themes emerged from our analysis of codeveloping a simulation on gender-sensitive care within vocational rehabilitation for youth with disabilities including (1) the relevance of gender within clinical practice and (2) creating a comfortable and safe space to enable gender-sensitive care (an overview of themes and subthemes is given in Multimedia Appendix 3).

### The Relevance of Gender Within Clinical Practice

During the first build session, most participants reflected on the extent to which gender was addressed within their clinical practice, ranging from hesitance to acknowledging but not talking about it to acknowledging and incorporating gender into practice.

#### Hesitant and Resistant

In the first build session, many participants resisted the idea that gender even mattered to their work. For example, one participant said:

I was sort of initially struggling with reflecting on this...I don’t think it is ever the first thing in my mind...I don’t think I necessarily have come across a blatant difference.

#2, simulation 1

Some participants hesitated at first about the possibility of gender differences and the role of gender within health care, despite learning about evidence of the importance of such differences. To illustrate, one health care provider reported:

I don’t see there’s a gender difference...I do not make any distinctions between genders.

#3, simulation 1

Others mentioned that they had never really considered the role of gender in their practice until participating in this study. Meanwhile, some participants stated that they considered gender within their practice but did not notice any gender-related patterns. For example, one said:

I don’t think about it enough systematically... I’m not sure I’ve ever noticed anything at the systems level surrounding gender; Or, that it’s ever been front of mind in any discussion with any families and youth.

#1, simulation 1

#### Gender Is Acknowledged But Not Openly Discussed

Some participants noted that gender was sometimes acknowledged within their practice but rarely explicitly discussed. For instance, one health care provider described, “at some level it may connect to gender. I’ve never brought that out into the open and I never had anyone else bring that into the open” (#2, simulation 1). Another health care provider concurred: “It’s interesting in the sense it’s being talked about, but not directly at the same time” (#5, simulation 1).

#### Gender in Clinical Practice

Some participants reported noticing gender differences (mostly binary) in their practice. For instance, a provider described, “over time we have possibly seen a slight increase in young men we’re serving in our programs” (#1, simulation 1). Another participant shared:

Autism, we do find in terms of diagnoses that females are diagnosed much older and later in life. Given a lot of the symptoms are masked...We’re seeing some differences there. With boys it seems a bit easier...the age of diagnosis is a bit younger...I’m just realizing that I am working mostly with males than females.

#3, simulation 1

Meanwhile, some participants mentioned gender differences around safety concerns and parental overprotection, particularly for female patients. To illustrate, one participant said:

I have families who will say, their experience outside of home and school the first things on their mind are around safety and vulnerability in the community...I can perhaps see a connection to gender because sometimes individuals have that concern more so for females.

#1, simulation 1

Furthermore, some participants noticed patterns in the career pathways of youth with disabilities, with females sometimes going into social sciences and males into computers. For example, a health care provider remarked: “I’m more often working with young men who are interested in computers and I don’t think I’ve had a young lady in my career who has said that’s what she’s dreamt of doing” (#4, simulation 1). The journal reflections indicated that building a simulation on gender-sensitive care helped encourage participants to share their reflections on this topic from an interprofessional perspective while also learning from each other.

### Creating a Comfortable and Safe Space to Enable Gender-Sensitive Care

The second main theme involved creating a comfortable and safe space to enable gender-sensitive care, which included patient-centered care, effective communication and rapport building, appropriate language and pronouns, respecting gender identity, and responding to therapeutic ruptures.

#### Patient-Centered Care

Participants highlighted the importance of applying patient-centered principles in providing gender-sensitive care. For example, one participant explained:

How we could enable people to hear and value and know the person first...sort of a person-centered start to a therapeutic relationship...We want to maximize their engagement.

#1, simulation 1

Another health care provider, who had some informal training in gender, explained how she also used a patient-centered approach to create a gender-sensitive environment:

People can easily sense when you’re uncomfortable; Or, you’re not sure how to approach the situation...It helps to open up the conversation as clinicians become more comfortable having a discussion and often times people don’t open up to others...Maybe comments and phrases people use that they don’t feel it’s a safe space...It comes from your authenticity and how you communicate that.

#5, simulation 1

Meanwhile, one participant shared how it might be helpful to use a patient-centered or solution-focused approach:

If they're a little harder to read it shows how we have to do some exploring...I would make that more open-ended, like in solution-focused coaching we ask what are your best hopes? This is what I’m here for...What are you hoping of getting out of meeting today? and giving more open-ended questions so it's giving space for that to come forward.

#2, simulation 2

#### Effective Communication and Building Rapport

Health care providers highlighted the importance of providing a comfortable and safe space that involved effective communication, developing rapport, listening, and understanding. Most participants agreed that developing rapport was the building block for effective communication. For instance, one participant said:

You have to establish rapport. So, the best, easiest way to do that from this perspective is to get people talking about themselves.

#6, simulation 2

Others agreed:

I don't think you're going to get anywhere without rapport.

#8, simulation 2

Another health care provider mentioned how to start the conversation:

Creating an open space where somebody can tell us their wishes and how to best support them...That lends itself to the conversation about gender...If we foster those conversations that were based on interests regardless of whether it is your son or your daughter...putting it in a neutral way...will give them the opportunity to explore this for themselves...having an atmosphere that doesn’t limit by gender.

#4, simulation 1

Participants described how their role involves getting patients to open up and they can do this by communicating effectively and building rapport. Others concurred that communication and rapport were important in providing gender-sensitive care. To illustrate, one health care provider shared:

In this whole process, we are probing for information. So, we are trying to poke holes at where can I connect with this person...trying to help them with their goal...and to get them to open up.

#10, simulation 2

#### Appropriate Language and Gender-Related Pronouns

Another part of creating a comfortable and safe space to enable gender-sensitive care involves using appropriate gender-related pronouns with patients. For example, one participant shared:

A lot of emphasis is being placed on gender and specifically on pronoun use and transgender. People in the program have shared lived experiences, and being misgendered...adds a lot of expectations that society might place on somebody thinking you’re female, but they might not identify as female.

#5, simulation 1

Participants described how exploring gender identity might play out while considering when they might ask patients about pronouns. For example:

It depends who you're working with, like this client also seems really shy and quiet so you wouldn’t want to be like, outright; but sometimes at the beginning, especially now, it’s happening a lot more; You can ask people their pronouns, on their forms, but it would obviously be super awkward. I would not recommend saying “oh never mind what are your pronouns?”; because that is kind of weird, but people are asking now what are your pronouns.

#7, simulation 2

One participant explained how they would respond if they had to deal with the issue of completing paper work and identifying a patient’s gender identity. They said:

I have to submit your information to the government in order for you to participate in our program. They have to check male or female or other. So, I see you checked male. When we interact together do you have a preferred name that is maybe different than your legal name? and a preferred pronoun, that may be different than what you wrote on this form? I have found that the individuals who are highly politicized we use language like “this is my identity; this is my pronoun. I don't identify as...my gender x.” They will use language like that.

#6, simulation 2

#### Respecting Gender Identity

Creating a comfortable and safe space involved participants respecting a patient’s gender identity including their values and preferences. Some participants described their experiences of working with gender-diverse patients and the potential challenges of misgendering a patient. To illustrate, one health care provider explained, “It’s trying really hard to be sensitive around identifying people with the way they see themselves and being respectful” (#5, simulation 1).

In the second simulation build session, several participants debated whether the gender identity of the patient was relevant. For instance, one participant said, “I wasn't sure when the youth (i.e., simulated participant) walked in whether or not I could put them into a category of male or female” (#9, simulation 2). Some participants commented that patients they know who are nonbinary are “used to people making assumptions, misgendering and so on. So, the best thing to do is catch it, as soon as you can” (#6, simulation 2).

Meanwhile, others described how it can be difficult to know the gender identity of a patient when there are often only binary choices (ie, male or female) on their chart, which may have been checked off by their parents when the child was originally registered at the hospital at a much younger age.

#### Awareness of Gender Stereotypes and Gender-Diverse Clients

Codeveloping a simulation on gender-sensitive care encouraged clinicians to share their reflections on this topic from an interprofessional perspective while learning from each other. For example, clinicians have demonstrated an awareness of gender stereotypes and gender-diverse clients. One clinician stated:

I’m certainly sensitive to it if I hear a parent try to compartmentalize their child...Oh, you’re a girl; you can’t possibly be a NASA space astronaut...hoping to not limit people based on gender.

#4

Others described the experience of working with gender-diverse patients and the potential problems of misgendering a patient. To illustrate, one said, “It’s trying really hard to be sensitive around identifying people with the way they see themselves and being respectful of that” (#5). Others have explained the potential problems of misgendering a client. For instance, a clinician explained:

Given that gender can be a big part of someone’s identity when someone has been misgendered based on how they look, you’ve kind of reduced their identity just to how they look and that makes conversations difficult.

#3

#### Responding to Therapeutic Ruptures

Participants told us how mistakes or “therapeutic ruptures” could happen as a result of a lack of knowledge about gender-sensitive care, particularly for patients who identify as gender diverse. A health care provider explained:

If you have made a mistake and maybe used the wrong pronoun, I think it’s how you create this space if you’re genuinely coming from the place of curiosity and wanting to learn. Then, often times in my experience people have been very receptive.

#5, simulation 1

Another shared:

Unexpectedness may be something that happens, if we have two major genders: male and female and someone brings us a new way of describing themselves and identifying themselves and having the language to engage and handle our own response in the professional and sensitive way...How to fix it, and how to do it right?

#3, simulation 1

Participants emphasized the importance of acknowledging any communication mistakes related to gender and working to rebuild a rapport with patients. Furthermore, providing gender-sensitive care involved recognizing and dealing with a potential unconscious bias. A health care provider shared:

Before we see a client, we look in the file. The first things we see are their name, age, gender, and diagnosis...Even noticing in my own practice we have lots of unconscious biases. I think, I’m going to see a sixteen-year-old boy with this diagnosis then I have a sense of this is what they’re going to be like. I do find myself not really exploring their gender identity if it’s already stated. However, if it said “other” I don’t have a lot of experience with, transgender female or male. I’d be more keen to explore, what is that like?

#5, simulation 1

In summary, participants described how important it was for them to acknowledge any mistakes or therapeutic ruptures related to gender identity.

## Discussion

### Principal Findings

Our study addressed an important gap in the literature by exploring how issues relevant to gender-sensitive care among vocational rehabilitation care providers within the context of building a simulation for people working with youth with disabilities. Focusing on gender-sensitive care is important because it is a key social determinant of health and a component of patient-centered care. Offering training in this area for health care providers could help to address health inequalities and optimize patient outcomes [46,69]. Our findings showed that the relevance of gender within clinical practice ranged considerably from hesitance to acknowledging it but not talking about it to incorporating gender into their practice. Research indicates that health care providers need an ongoing awareness of their values, assumptions, and beliefs and how these relate to their patients, which may be developed through reflective practice [53,70]. Furthermore, studies also show that having knowledge about gender is often associated with more positive attitudes and enhanced patient care [26,71,72].

Our findings highlight that creating a comfortable and safe space is a salient aspect of gender-sensitive care. In particular, we found the following elements to be important for creating a safe space: patient-centered care, effective communication and rapport building, appropriate language and pronoun use, respecting gender identity, and responding to therapeutic ruptures. These findings are consistent with culturally sensitive care models that emphasize the importance of self-awareness and sensitivity to one’s own values, biases, and power differences with patients while also understanding their values and priorities [73,74]. Other research demonstrates the importance of the awareness of health care providers of any biases they have because it could influence health care decisions [75].

Our findings also revealed that addressing therapeutic ruptures regarding gender, and particularly misgendering someone, was an important aspect of gender-sensitive care. Our results are consistent with other research highlighting that failing to identify a patient by affirming their name and pronoun within a medical setting can impact patient satisfaction and quality of care, particularly for LGBTQ+ patients [76]. Gender-related therapeutic ruptures could be a result of binary documentation (ie, male or female-only options), which could potentially misgender a patient, causing stress or stigmatization [28]. It is important that health care providers address gender diversity in their clinical practice. The World Health Organization advocates that sex and gender are addressed both within and outside of health care [31].

Our results highlight that effective communication is an important component of gender-sensitive care. This finding aligns with the principles of patient-centered care and culturally sensitive care, which require health care providers to be open to information from patients about their needs, expectations, and preferences while listening and respecting the patients [77]. Although many clinicians are trained in patient-centered care, it may be worthwhile to have more specific training in gender-sensitive care. Research indicates that communication plays a critical role in therapeutic sessions, ongoing patient-clinician relationships, and patient satisfaction [77,78]. Effective communication could help health care providers to better understand patients’ needs and priorities, which could help them to tailor their recommendations [77].

Most of our findings focused on issues related to gender identity and, to some extent, gender roles; however, there was little discussion of gender relations or institutionalized gender (eg, power relations between men and women) [25]. It is important to recognize that gender is part of a larger sociopolitical and cultural context and that health care organizations themselves are gendered [26]. Surprisingly, few participants reflected on how their own gender could potentially impact the development of rapport with patients. Further unpacking this trend is important because most Canadian health care providers in pediatric rehabilitation are female, whereas most pediatric rehabilitation patients are male [79]. In addition, recognizing gender differences within the health care context along with understanding the role of one’s own gender within professional practice are critical for avoiding and reinforcing gender stereotypes [29,80,81].

Participants in our study had the opportunity to reflect on their own skills in gender-sensitive care. Building a simulation (Multimedia Appendix 1) allowed participants to reflect on this topic within an interprofessional setting while listening to, and learning from, other participants and researchers. Participants identified gender-sensitive approaches by sharing experiences and perspectives regarding gender within their practice within the format of building a professional development simulation, which created an opportunity for participants to work directly with live simulated participants. Our study is novel in that few studies have involved simulation facilitators in the process of building a simulation but instead rely on predeveloped character roles [49]. Other research shows that using patient narratives is a useful tool for reflection among health care providers [82]. Our findings were consistent with other studies indicating that interprofessional discussions of case scenarios helped to broaden individual perspectives from different professions within a safe environment [77].

### Limitations

Our study has some limitations that are important to acknowledge. First, the majority of the participants in our study were women; however, this gender-unbalanced sample reflects the gender composition of health care providers within pediatric rehabilitation (ie, majority are women) [58,83]. Second, we used a small, qualitative sample drawn from one city. Thus, further testing with a larger sample and long-term outcomes (ie, changes in knowledge and practice) are needed. Future studies should consider evaluating the impact of the simulation on practices of health care providers. Third, the participants who chose to participate in our study may have had more of an interest in or experience with disability and were not representative of all pediatric rehabilitation health care providers. In addition, it is also important to recognize that gender norms and values often vary by culture, which is an area of further exploration.

### Conclusions

This study explored perspectives of health care providers on relevant issues to provide gender-sensitive care within pediatric rehabilitation within the context of building a professional development simulation tool. Our findings showed two key themes, including the relevance of gender within clinical practice and creating a comfortable and safe space (ie, practicing patient-centered care, effective communication and rapport building, respecting gender identity and being aware of stereotypes, and responding to therapeutic ruptures) to enable gender-sensitive care. Future research should consider exploring the impact that simulations may have on behaviors in clinical practice.

## Appendix

Multimedia Appendix 1 Simulation scenario template.

Multimedia Appendix 2 Focus group discussion guide.

Multimedia Appendix 3 Table overview of themes.

## Abbreviations

LGBTQ+: lesbian, gay, bisexual, transgender, queer, and other sexual identities
