# Supplementary material for: Exploration of Gender-Sensitive Care in Vocational Rehabilitation Providers Working With Youth With Disabilities: Codevelopment of an Educational Simulation
Source: JMIR Form Res. 2021 Mar 15;5(3):e23568. doi: 10.2196/23568 (PMC8075068; doi:10.2196/23568)
Supplement: Multimedia Appendix 1 [file formative_v5i3e23568_app1.docx]

**Multimedia appendix 1. Simulation scenario template**

**Issues to Explore**Building rapport to allow exploration of gender identification, assumptions and bias that impact patient-centred career discussions

**Learning Objectives**

- Explore rapport building strategies that allow open conversation and full understanding of who the client is and how they want to be seen and understood (Understand how whole person is relevant to rehabilitation)
- Employs effective communication to repair unexpected missteps in the relationship (therapeutic rupture and bias) (encouraging self-discovery vs assumption driven)
- Explores issues of gender identity and personal details in a safe, compassionate manner **(**How to engage someone to share what’s most important to them; Reflect on person focus identity strategy; rapport building enhance outcomes)

**Instructions for the Learner**

You are an experienced clinician (e.g., social worker, occupational therapist, life skills coach, etc.) working in patient-centered and solution-focused framework. You are about to meet Francis to explore and discuss preparing them for transition to adulthood (e.g., employment opportunities). Francis is an existing client at the hospital, having received physiotherapy and occupational therapy last year. Their file indicates a 17-year-old male with an acquired brain injury, who was referred to you and is meeting you for the first time. Please meet Francis and explore interests and next steps to prepare for their future.
