# Supplementary material for: Exploration of Gender-Sensitive Care in Vocational Rehabilitation Providers Working With Youth With Disabilities: Codevelopment of an Educational Simulation
Source: JMIR Form Res. 2021 Mar 15;5(3):e23568. doi: 10.2196/23568 (PMC8075068; doi:10.2196/23568)
Supplement: Multimedia Appendix 2 [file formative_v5i3e23568_app2.docx]

**Multimedia Appendix 2. Journal template and focus group discussion questions**

**Journal template** (adapted from Zubarai et al. 2016)

Participant __ Date ___ Session ____

- How do you think today’s build session will affect your communication skills regarding disability disclosure with clients you work with?
- Anything else you would do differently after today’s session?
- Reflect on the previous session and whether you have any specific learning goals for today.

**Focus group discussion guide** (Adapted from Zubairi et al. 2016)

1. In what ways has participating in this study shaped your thinking about gender-sensitive care?
2. Were there any ‘aha moments’ that you encountered during the process of building the simulation? (Probe for real-life situations that the simulation reminded them of)
3. For you, what are the important aspects of gender sensitive care?
4. What strategies have you developed / learned to enhance gender-sensitive care?
5. How might you change your communication the next time you are in a situation where you think someone’s gender may affect the interaction?
6. What is the utility of having a stakeholder representative available during the learning process? What are the drawbacks, if any?
7. What do you see as the value of building the simulation in promoting the development of your communication skills with clients you work with?
8. What factors in the process of building a simulation promote the development of gender-sensitive care skills?
9. What factors in the process of building a simulation inhibit the development of communication skills?
10. Is there anything else that you’d like to add about your experience of participating in the development of the simulation?
