# Supplementary material for: Exploration of Gender-Sensitive Care in Vocational Rehabilitation Providers Working With Youth With Disabilities: Codevelopment of an Educational Simulation
Source: JMIR Form Res. 2021 Mar 15;5(3):e23568. doi: 10.2196/23568 (PMC8075068; doi:10.2196/23568)
Supplement: Multimedia Appendix 3 [file formative_v5i3e23568_app3.docx]

**Multimedia appendix 3. Table overview of themes and sub-themes**

| **Themes** | **Sub-themes** |
| --- | --- |
| Relevance of gender within clinical practice | -Hesitant and resistant  -Gender is acknowledged but not openly  discussed  -Gender within clinical practice |
| Creating a comfortable and safe space to enable gender-sensitive care | -Patient-centred care  -Effective communication and building rapport  -Appropriate language and gender-related pronouns  -Respecting gender identity  -Awareness of gender stereotypes and gender diverse clients  -responding to therapeutic ruptures |
